# Bacterial capsules: Occurrence, mechanism, and function

**DOI:** 10.1038/s41522-024-00497-6

**Published:** 2024-03-13

**Authors:** Shuji Gao, Wenjie Jin, Yingying Quan, Yue Li, Yamin Shen, Shuo Yuan, Li Yi, Yuxin Wang, Yang Wang

**Affiliations:** 1 College of Animal Science and Technology, Henan University of Science and Technology, Luoyang, 471000 China; 2 Henan Provincial Engineering Research Center for Detection and Prevention and Control of Emerging Infectious Diseases in Livestock and Poultry, Luoyang, 471003 China; 3 College of Life Science, Luoyang Normal University, Luoyang, 471934 China

**Keywords:** Biofilms, Bacteriology, Pathogens

## Abstract

In environments characterized by extended multi-stress conditions, pathogens develop a variety of immune escape mechanisms to enhance their ability to infect the host. The capsules, polymers that bacteria secrete near their cell wall, participates in numerous bacterial life processes and plays a crucial role in resisting host immune attacks and adapting to their niche. Here, we discuss the relationship between capsules and bacterial virulence, summarizing the molecular mechanisms of capsular regulation and pathogenesis to provide new insights into the research on the pathogenesis of pathogenic bacteria.

## Introduction

In bacteria, polymers known as capsules are generated at the periphery of the cell wall, enveloping the entire cell. Capsules connect to the peptidoglycan in Gram-negative bacteria or the plasma membrane in Gram-positive bacteria via covalent attachments to either phospholipid or lipid-A molecules. Capsules may also establish direct connections with surface proteins on the bacterial membrane^1^. For most bacteria, capsules primarily consist of polysaccharides, exemplified by *Streptococcus suis* (*S. suis*)^2^. Some capsules primarily consist of polypeptides, as in *Bacillus anthracis*^3^, while others, like *Bacillus megaterium*^4^, contain both polysaccharides and polypeptides. Capsular polysaccharides (CPS) were initially described as “halo” by Pasteur in 1881. CPS was isolated and discovered by Avery and Dochez in 1917^5^. It was not until 1925 that Avery elucidated the carbohydrate nature of the substance in the microbial capsule^6^. Capsules are prevalent in natural pathogens and participate in various bacterial cellular processes. Capsules regulate the size and dispersion of bacterial biofilm, contributing to sustained infections within hosts^7^, reducing the efficacy of antimicrobial peptides and complement^8^, suppressing phagocytosis by innate immune cells, promoting intracellular survival^7,9^, and aiding in defence against antimicrobial agents^10^. In this review, we summarize the role of capsules in bacterial virulence and analyze their regulation mechanisms.

## Capsules biosynthesis

Capsules primarily consist of high molecular weight polysaccharides, which are essentially oligosaccharide repeating units. However, some bacterial capsules are atypical: the capsule of *Yersinia pestis* is a protein polymer composed of 17-kDa subunits^11,12^, and the capsule of *Bacillus anthracis* consists of D-glutamic acid^13^. Additionally, the O-antigen capsule and the capsule-like complex (CLC) are identified as capsules of *Francisella tularensis* (*F. tularensis*)^14^. The O-antigen capsule is composed of mannose, rhamnose, and dideoxy sugars, whereas the CLC comprises proteins and carbohydrates^15^. However, the precise role these capsules play in the virulence of *F. tularensis* remain to be fully elucidated. Further research is required to clarify the structure and contributions of each capsule to the pathogenesis and virulence of *F. tularensis*.

**Fig. 1:**
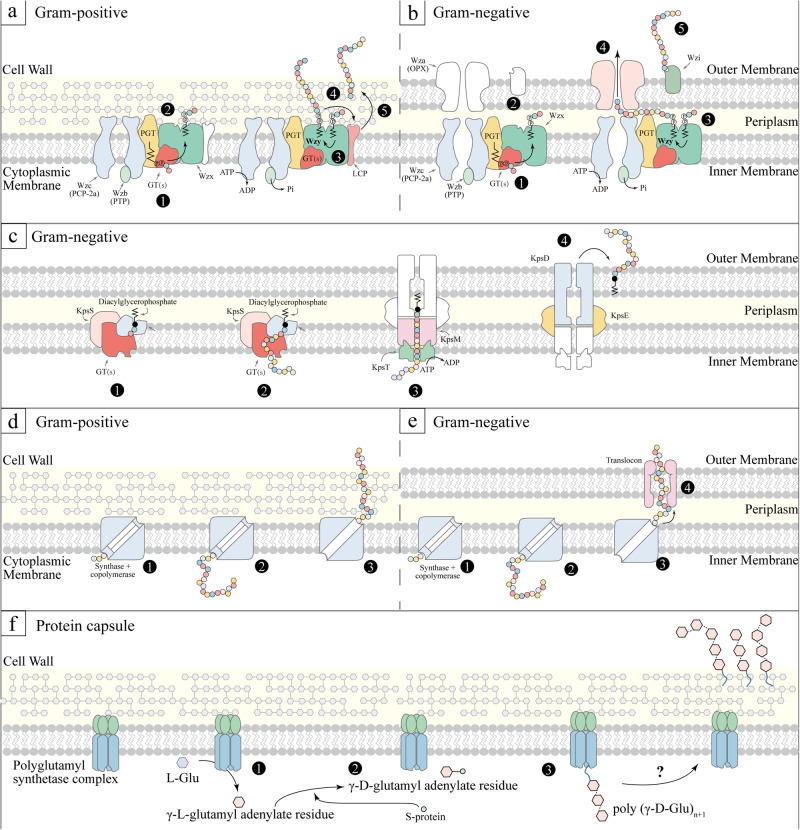
Capsule synthesis mechanisms. Wzy-Dependent mechanism. The first three steps are universal: ①, phosphoglycosyltransferase (PGT) and serotype-specific glycosyltransferases (GT(s)) synthesize undecaprenyl diphosphate-linked oligosaccharide repeat units; ②, Wzx facilitates the translocation of these units across the membrane, followed by Wzy-mediated polymerization; ③, The phosphorylation cycle of Wzc, catalyzed by Wzb, is a crucial step. In Gram-positive bacteria (**a**): ④, The newly formed polymer is transferred onto a peptidoglycan assembly intermediate; ⑤, It gets anchored to the cell wall through LCP activity. In Gram-negative bacteria (**b**): ④, The polymer translocates across the outer membrane via Wza; ⑤, In some prototype and other species, Wzi assists in organizing the translocated polymer into surface-associated capsule structures. **c** ABC Transporter-Dependent mechanism. For Gram-negative bacteria, ①-② involve the assembly of sugar chains; ③, the ABC transporter (KpsMT) plays a pivotal role in exporting these chains; ④, KpsE and KpsD are essential for translocating the assembled chains across the periplasm and outer membrane. The specifics of the ABC-dependent pathway in Gram-positive bacteria remain unclear. Synthase-Dependent mechanism: ①, Initial synthesis of the short sugar chains; ②, Extension of these chains by glycosyltransferases. In Gram-positive bacteria (**d**): ③, Transfer of the polymer to the outer membrane. In Gram-negative bacteria (**e**): ③, Polymer translocation and anchoring to the cell wall via a translocation anchor. (**f):** Protein capsule synthesis pathway. The synthesis of the poly (y-D-glutamyl) capsule in *Bacillus licheniformis* involves a series of membrane-associated enzymatic reactions. Here, a polyglutamyl synthetase complex catalyzes the activation, racemization, and polymerization of L-glutamic acid, resulting in a high molecular weight polymer of y-D-glutamic acid. ① L-Glu + ATP ⇌ γ-L-Glu-AMP + PPi; ② γ-L-Glu-AMP + S-protein⇌ γ-L-Glu-S-protein + AMP; γ-L-Glu-S-protein ⇌ γ-D-Glu-S-protein; ③, γ-D-Glu-S-protein + poly (γ-D-Glu)_n_ → poly (γ-D-Glu)_n+1_ + S-protein. GT glycosyltransferase, LCP LytR-CpsA-Psr, OPX outer membrane polysaccharide, PCP polysaccharide copolymerase; PGT, phosphoglycosyltransferase, PHP polymerase and histidinol phosphatase, SA polysaccharide A, PTP phosphotyrosine phosphatase, Glu glutamate, S-protein a protein-bound thioester, as a second intermediate for the growing polymeric chains.

Currently, three primary capsule synthesis pathways are recognized: the Wzx/Wzy-dependent mechanism, the ATP-binding cassette (ABC) transporter-dependent mechanism, and the synthase-dependent mechanism^16,17^. Over 90% of *S. pneumoniae* serotypes synthesize capsules via the Wzx/Wzy-dependent mechanism, characterized by the formation of repeat units and nonprocessive polymerization^18^. This mechanism is also fundamental to the synthesis of group I and group IV capsules in Gram-negative bacteria^19^. The initial step involves transferring a 1-phosphate to the lipid carrier (undecyl isoprene phosphate) on the cell membrane’s cytoplasmic surface. Following this, the complete repeat unit is turned outward by the Wzx flip enzyme, and the Wzy polymerase attaches the growing polymer chain to the newly formed repeat unit (Fig. 1, a, b)^19^. In *S. pneumoniae* serotypes, both with and without glucose (Glc), this step is accomplished by liposome and Glc-1-phosphotransferase (Glc-1-P) CpsE/WchA and CPS site transferase from Wcil, WcjG or WcjH homologous groups, respectively^20^. Group II and group III capsules are called ABC-dependent capsule. In this pathway, new polysaccharide chains undergo polymerization in the cytoplasm and are associated with phospholipid receptors. These chains are then transported across the intima by ABC transporters (Fig. 1c). Even with these variations, both Wzx/Wzy and ABC-dependent mechanisms employ similar outer membrane proteins belonging to the polysaccharide export family for the transportation of capsules across the bacteria’s outer membrane^16^. For group IV capsules, CPS synthesis relies on Wzy polymerase and does not involve the Wzx flip enzyme. Additionally, CPS synthesis can occur via the synthase-dependent mechanism, wherein specific enzymes are responsible for initiating, polymerizing, and translocating the capsules (Fig. 1, d, e)^21^. For instance, *S. pneumoniae* serotype 3 and 37 utilize a single enzyme mechanism, which initiates capsule synthesis by transferring sugar to lipid receptors and subsequently adding additional sugars for extension^22^. However, it remains a puzzle why the Wzx/Wzy-dependent mechanism predominates in most Gram-positive bacteria, while the synthase-dependent mechanism is less common^23^.

The initial identification of protein capsules occurred in *Bacillus anthracis*^24,25^, and subsequent elucidation of their biosynthetic pathways was achieved in *Bacillus licheniformis*^26^. In the absence of hydroxylamine, γ-L-glutamyl adenylate residue forms, which is then converted to γ-D-glutamyl adenylate residue upon activation. A protein-bound thioester (S-protein) may serve as a second intermediate in this process^27^. The final step involves transferring the activated glutamate to an endogenous membrane-bound poly (γ-D-glutamyl) acceptor. Consequently, polyglutamyl chain extension occurs by adding new glutamyl units to the terminal amino group of the receptor-bound glutamyl residue (Fig, 1f). In summary, gaps remain in understanding protein capsule synthesis and assembly, particularly regarding specific molecular mechanisms, such as the completed polypeptide chain outside the membrane.

## Serotypes and pathogenicity

**Fig. 2:**
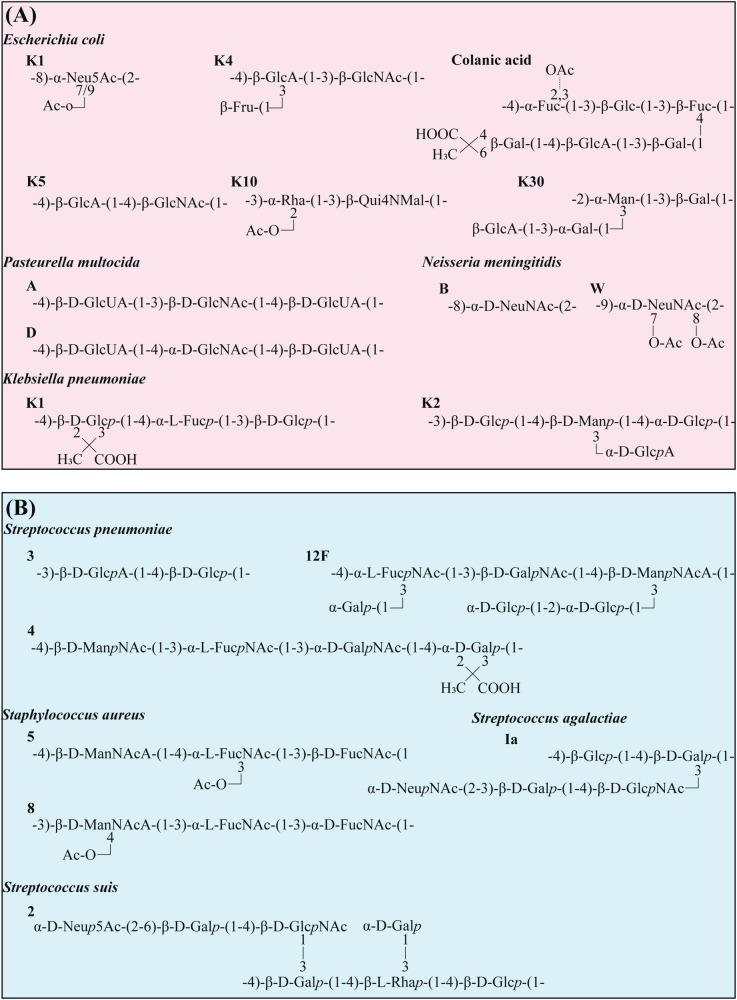
Bacterial capsule structure. **A** Gram-negative bacteria. **B** Gram-positive bacteria. Glc, glucose; GlcA (GlcUA), glucuronic acid; Gal galactose, Fuc fucose; Fru fructose; Man mannose; Rha, rhamnose; GlcNAc N-acetylglucosamine; ManNAc, N-acetylmannosamine; GalNAc, N-acetylgalactosamine; ManNAcA, N-acetylmannosaminuronic acid; FucNAc, N-acetylfucosamine; NeuNAc (Neu5Ac), N‑Acetylneuraminic Acid (sialic acid); Qui4NMal, 4-(2-carboxyacetamido)-4,6-dideoxyglucose; *p*, phosphate; Ac, acetate; The structures have been published elsewhere: *Escherichia coli* (K1, K4, K5, K10, K30, colanic acid)^19^; *Pasteurella multocida* (A, B)^207^; *Neisseria meningitidis* (B, W)^132^; *Klebsiella pneumoniae* (K1, K2)^208,209^; *Streptococcus pneumoniae* (3, 4, 12F)^17^; *Streptococcus agalactiae* (Ia)^209^; *Staphylococcus aureus* (5, 8)^210^; *Streptococcus suis* (2)^211^.

Capsule types are influenced by various factors, including the number and sequence of monosaccharide components, the position of glycosidic linkages, the configuration (L or D) of components, and the degree of chemical modification, such as O-acetylation. Consequently, capsular structures are diverse, including hyaluronic acid (HA)^28^, heparosan^29^, chondroitin^30^, polysialic acid (PSA)^31^, and colanic acid^32,33^. The composition of the capsules facilitates further differentiation of bacteria into distinct groups (serotypes, serovars). *Escherichia coli* (*E. coli*) produces approximately 80 distinct capsule types, which were further categorized into four major groups: Group I, Group II, Group III, and Group IV, including the PSA-containing K1 capsule, chondroitin-containing K4 capsule, and heparosan-containing K5 capsule (Fig. 2A)^34^. Colanic acid is a capsule whose structure is similar to the group I capsule. Its biosynthesis occurs via the Wzx/Wzy-dependent pathway as in the the group I capsule^35^. A notable distinction is that *E. coli* cultured in laboratory conditions at 37 °C does not produce colanic acid^36^. In *S. pneumoniae*, 93 capsular serotypes have been identified, most of which are capable of causing infection^18,37–39^. *S. pneumoniae* primarily synthesized its CPS in trisaccharide units, though variations exist among serotypes. For serotype 12 F, CPS synthesis is based on hexasaccharides^40^. In serotype 4, CPS comprises a tetrasaccharide repeating unit with pyruvate modifications (Fig. 2B)^41^. Serotype 6 A’ CPS, based on tetrasaccharides, includes a rhamnose modification^42^. *Streptococcus suis* (*S. suis*) strains were initially classified into 35 serotypes based on CPS antigenicity^43–45^, later revised to 33 serotypes^46^.

Significant variation in capsule structure exists between different bacteria and among serotypes within the same species, contributing to diverse virulence (Table 1). Specific *S. pneumoniae* serotypes (1, 4, 5, 8, 12F, 18C and 19A) are highly invasive, while others (6A, 6B, 11A, and 23F) are reported to be less aggressive^47–50^. In *S. aureus*, serotypes 5 and 8, out of 11 known serotypes, are predominantly responsible for human infections^51^. The polysaccharides of serotypes 5 and 8 differ only in sugar linkages and O-acetylation sites of the mannosaminuronic acid residues (Fig. 2B)^52,53^. In *Haemophilus influenzae* (*H influenzae*), the capsules composed of polyribose ribitol phosphate connected by phosphodiester bonds render serotype b (Hib) the most virulent, followed by serotype a (Hia) and other capsular types^54^. *Streptococcus pyogenes* (GAS) groups A-C causing pharyngitis are typically not associated with skin infections (group D), and the opposite is also true, although certain serotypes show no particular tissue preference (group E)^55,56^. Considering these observations, researchers speculate that capsule virulence may be correlated with the frequency of monomer repetition or the specific type of monomer. In a mouse infection model, highly virulent serotypes of *Klebsiella pneumoniae* (*K. pneumoniae*), notably K2, which is associated with bacteremia, are devoid of the mannose-α-2/3-mannose structure that present in less virulent strains^57,58^.

**Table 1:** Capsular-related bacterial diseases

Species	Capsule type	Diseases	Function	Refs
**Gram-negative bacteria**				
*Escherichia coli*	K1, 4	Sepsis	Immune evasion	^85,177,178^
	K1	Meningitis	Invasion	^103^
	K1	Spontaneous bacterial peritonitis	Translocation	^179^
	K2, K5	Urinary tract infections; Ulcerative colitis	Adhesion	^180,181^
*Pseudomonas aeruginosa*		Sepsis; Septic shock	Immune evasion	^182^
*Klebsiella pneumoniae*	K1, K1, K2, K16, K28, K57, K63	Bacteremia; liver abscess	Immune evasion	^183,184^
	K2, K1, K57, K5, K20, K54	Meningitis	Serum resistance; Biofilm formation	^185,186^
	K20	Burn infection	Unknown	^58^
	K1	Urinary tract infections	Biofilm formation; Invasion	^187^
**Gram-positive bacteria**				
*Streptococcus pneumoniae*	6B	Meningitis	Immune evasion	^188^
	1, 2, 4 and 9V	Pneumonia	Immune evasion	^189,190^
		Endocarditis	Adhesion	^191^
	2, 4, 6B, 7F	Meningitis	Immune evasion; Translocation	^190,192–194^
	3, 6, 9, 15, 19, 23	Acute conjunctivitis; Endophthalmitis	Immune evasion	^195,196^
	4, 3, 19A	Respiratory tract infection; Acute otitis media	Adhesion	^78,197^
*Staphylococcus aureus*	5, 5	Bacteriemia; Atopic dermatitis	Adhesion; Immune evasion	^198,199^
*Streptococcus pyogenes*	M18	Pharyngitis	Immune evasion	^200^
		Toxic shock syndrome	Immune evasion	^201^
*Streptococcus suis*	2, 14	Meningitis	Anti phagocytosis; Adhesion	^202–204^
	1	Polyarthritis	Immune evasion	^205^
*Bacillus anthracis*	Poly-γ-D-glutamic acid capsule	Septicemia	Immune evasion	^3,206^

Capsular switching is common in bacterial populations, exemplified by *S. pneumoniae* serotypes 11A and 11E. Serotype 11E, while rare in nasopharyngeal (NP) isolates, is frequently found in invasive pneumococcal disease (IPD) isolates^59^. Serotype 11E is identical to 11A, except that *wcjE* is inactivated, resulting in a lack of binding to ficolin-2^60,61^. In addition, most serotype 11E isolates possess *wcjE* mutations, including missense and nonsense mutations, single-base insertions and deletions, as well as transposon insertions^61^. This indicates that serotype 11A evolves into serotype 11E within populations, facilitating escape from ficolin-2-mediated phagocytosis during invasive *S. pneumoniae* infections. Similarly, the distinction between serotypes 9V and 9A lies solely in *wcjE* mutations, with the inactivation supporting the evolution from 9V to 9A^62,63^. Frequent variations through homologous recombination and horizontal gene transfer result in numerous uncharacterized components within capsular systems, including genetic components, proteins, or other molecular structures^64^. As a result, only a limited number of studies have reported on the frequency and diversity of capsules. To address this, Rendueles et al. developed protein profile models for identifying key components of various capsule biosynthetic pathways^65^. Wick et al. introduced Kaptive Web, an online tool for rapidly typing *Klebsiella*’s K and O loci^66,67^. This method was also applied to identify *Acinetobacter baumannii* (*A. baumannii*)’s KL and OCL loci^68^. These developments offer significant technical support for identifying prokaryotic capsules.

## Biological functions and pathogenicity of capsules

### Adherence

**Fig. 3:**
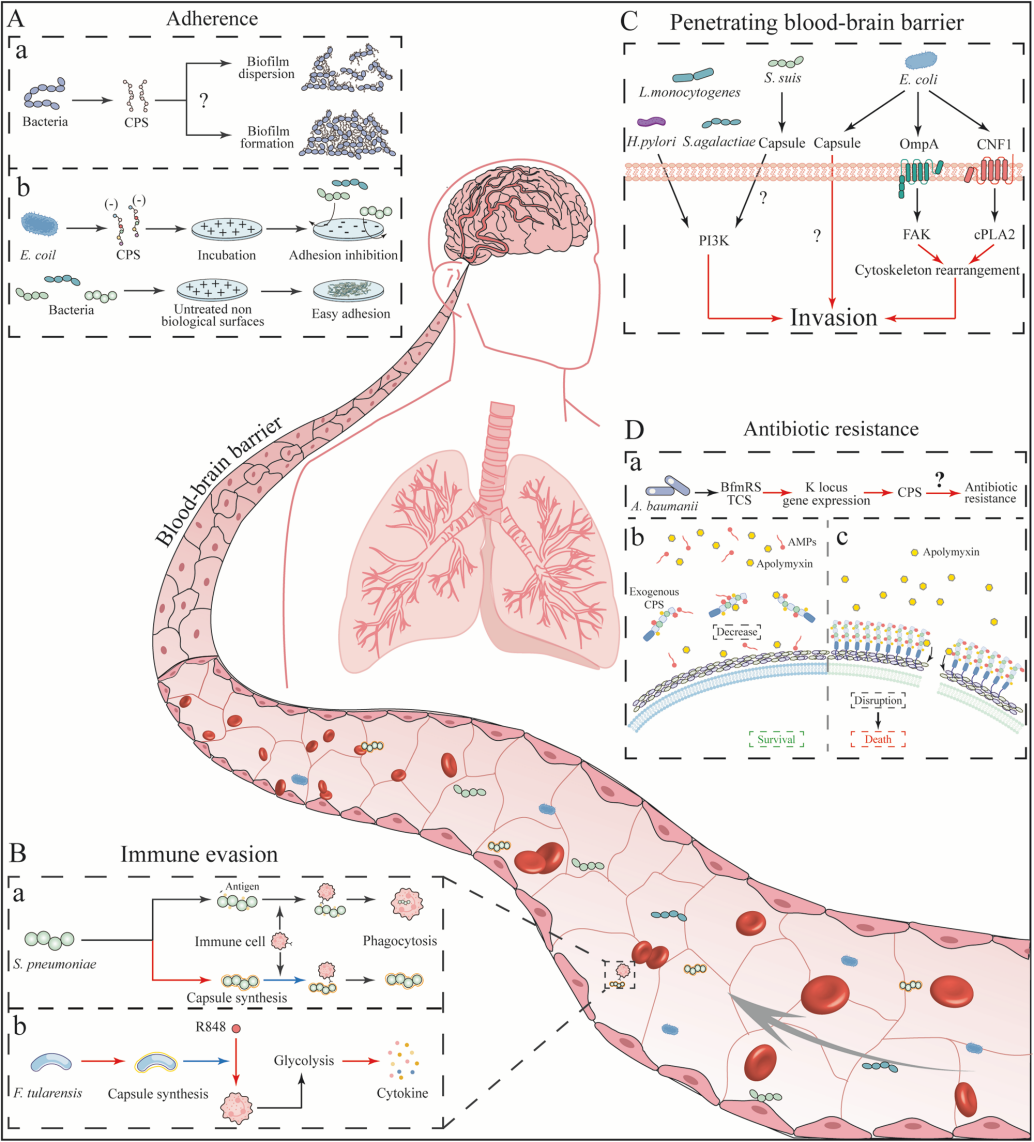
The functions of capsule in the process of pathogenic bacterial infection. **A** The influence of capsules on bacterial adherence. (a) Capsules promote or inhibits the formation of bacterial biofilm. (b) The addition of exogenous CPS changes the physical properties of the surface of non-biological materials, make them carry high negative charge and inhibit the adhesion of bacteria. **B** Interfering with host immune responses. (a) *S. pneumoniae* masks body surface antigens through capsule to avoid phagocytosis by macrophages. (b) *F.tularensis* inhibits the metabolic transformation of phagocytes through the synthesis of capsules, and finally inhibits the secretion of cytokines. **C** Assisting bacterial BBB penetration. Bacteria break through the blood-brain barrier by stimulating actin cytoskeleton rearrangement and various signal transduction pathways, including phosphatidylinositol-3-kinase (PI3K) and cytoplasmic phospholipase A2 (cPLA2). But at present, the effect on capsules is only phenotypic. **D** The impact of capsules on antibiotic resistance. (a) *A. baumanii* regulates the expression of K locus gene through the BfmRS TCS, which further promotes the synthesis of CPS and resists the killing of antibiotics, but the mechanism of CPS is still unknown. (b) Exogenous CPS adsorbs antimicrobial peptides (AMPs) and polymyxin, thus promoting the survival of bacteria. (c) The CPS located on the surface of bacteria adhere to polymyxin, which promotes the cleavage of polymyxin to the bacterial cell wall and finally promotes the death of bacteria. Positive regulation is indicated by red arrow, and negative regulation by blue arrow.

Capsules facilitate bacterial adhesion to surfaces and other bacteria, enhancing colonization in diverse niches and fostering biofilm formation. The bacterial biofilm matrix consists of polysaccharides and is enriched with extracellular proteins and various small molecules, including extracellular DNA (eDNA)^69^. Eliminating the *frwC* gene, encoding a hypothetical fructose-specific enzyme II C, can stimulate *magA* (also known as *wzy*, encoding a polymerase essential for capsule synthesis) transcription, thus enhancing CPS production and in vitro biofilm formation in *K. pneumoniae*^70^. Self-phosphorylating Wza proteins facilitate CPS assembly. The absence of *wza* impairs CPS transport to the outer membrane and its fixation, markedly diminishing cell adhesion in *A. baumannii* and leading to in vitro biofilm defects^71^. There is also a mechanism different from the “classic” static biofilm formation. During periods of high CPS expression, floating *S. pyogenes* aggregates connect via CPS to facilitate in vitro biofilm formation (Fig. 3A, a). However, the “classic” mechanism can overshadow this biofilm formation mechanism^72^. Biofilm resulting from bacterial attachment can have far-reaching effects. For example, the biofilm formation of retention tubes in inpatients will cause more severe nosocomial infections^73^. The *Pseudomonas aeruginosa* biofilm in patients with cystic fibrosis forms a permeable barrier that can resist the function of antibiotics^74^.

Nevertheless, the impact of capsules on bacterial adhesion remains a subject of debate. An alternative perspective posits that capsules formation primarily occurs during the mature stage of bacterial biofilm, where CPS secretion facilitates biofilm dissociation (Fig. 3A, a), enabling bacterial dispersion and subsequent biofilm development^7^. This effect could be due to the masking of adhesive proteins on the bacterial surface or the capsule’s physical and chemical properties. Antigen 43 (Ag43), a surface protein facilitating cell-to-cell aggregation^75^, is hindered in its function when a K1 or K5 capsule is expressed, as the extended polysaccharides sterically block adhesion^76^. Likewise, CPS production can obscure ClfA (Clumping Factor A) on the cell wall, thereby reducing *S. aureus*’s capacity to adhere to platelets^77^. Additionally, certain studies indicate that capsules may negatively regulate initial in vitro biofilm formation. The negative charge carried by CPS is thought to hinder early bacterial accumulation, consequently inhibiting in vitro biofilm formation^78^. This phenomenon, far from being accidental, has garnered attention since the early 21st century, with the capsule’s biological properties considered promising for medical surface coatings. Group II capsules extract of *E. coli* can induce a significant charge reversal at latex particles’ interface ζ (zeta) potential, resulting in a highly anionic nature^79^. Besides electrostatic modifications, active supernatants can also remodel colloid surface properties, potentially involving surface hydration and steric repulsion. Consequently, Group II capsules significantly reduce biofilm formation on glass surfaces by *E. coli* and various Gram-positive and Gram-negative pathogens, primarily by weakening cell-surface contacts and reducing cell-cell interactions (Fig. 3A, b).

The influence of capsule serotypes on in vitro biofilm varies. In *Bacteroides thetaiotaomicron* biofilm grown in a chemostat for 8 days, CPS 8 expression was upregulated, whereas capsules 1, 3, 4, and 6 showed downregulated^80^. However, there is no evidence that increased in vitro adhesion correlates with improved colonization in axenic mice, indicating that enhanced in vitro biofilm formation may not predict in vivo colonization capacity^81^. Capsules-mediated bacterial adhesion is also linked to the bacterial growth cycle. *S. aureus* exhibits stronger adhesion to human endothelial cells (EC) during the exponential growth phase compared to the stationary phase^82^. Ashbaugh et al. conducted comparative experiments with primate models and revealed that CPS-free mutants were eliminated from the baboon pharynx more rapidly than wild-type strains in short-term colonization studies^83^. In long-term carrier models, it was observed that carriage isolates developed mutations leading to reduced or absent hyaluronic acid production, suggesting that CPS enhances transmission and initial colonization. Additionally, the late downregulation or loss of CPS synthesis may aid in the long-term survival of the strain in vivo^84^.

### Resistance to host immunity

In invasive bacterial infections, the capsule’s interaction with the host immune system is pivotal in determining infection outcomes^85^. Capsules confer resistance to nonspecific host defense mechanisms, particularly without specific antibodies. Such mechanisms involve the activation of the complement cascade and C3b-mediated neutrophil phagocytosis via alternative pathways. Activation of the alternative pathway occurs via the nonspecific attachment of the serum protein C3b to the bacterial surface. Upon attachment, C3b engages with factor B, forming C3 convertase (C3bBb). This leads to enhanced C3 attachment and the development of a membrane attack complex (MAC) on the bacterial surface, culminating in lysis and the demise of the bacteria^86^. CPS impedes the binding of immunoglobulin G (IgG) to bacteria by masking antigen-binding sites on the bacterial surface and diminishes the effect of C3b/iC3b by obstructing the conversion of C3b to iC3b on the bacterial surface (Fig. 3B, a)^87^. CPS that contains N-acetylneuraminic acid do not initiate the alternative pathway. The inhibition of this pathway can be ascribed to the direct interaction of N-acetylneuraminic acid in polysaccharides with factor H. The bound factor H serves as a cofactor, initiating the combination of factor I and C3b to form iC3b, thus preventing MAC formation^88^. The capsule collaborates with cell surface structures like O antigen to resist complement-mediated killing. Consequently, this specific combination exhibited by the bacteria confers significant resistance to complement-mediated killing^89^. Additionally, CPS can counteract complement-mediated opsonophagocytosis. The capsules mask C3b on the inner cell surface, thereby preventing its binding to the C3b receptor (e.g., CR1) on the phagocyte surface^90,91^. Hurst et al. noted in their immunohistochemical study of a mouse infection model that neutrophils infiltrated the core of GAS infections, irrespective of HA capsule expression. Within 24 h post-infection, there was an upsurge in the neutrophil population (GR1+), and strains with capsular defects were rapidly eliminated by 48 h. Moreover, this does not lead to nasopharyngeal restructuring conducive to favorable inflammation. Furthermore, capsules may play a role in pathogenesis by preventing the entrapment of *S. pneumoniae* in neutrophil extracellular traps^92^.

Beyond passive responses to the host immune system, bacterial capsules actively modulate host immune responses by directly influencing cytokine release and disrupting the coordination of host cell-mediated immune responses^93^. For instance, the capsule of *Francisella tularensis* (*F. tularensis*) can impede the R848-induced increase in lactic acid secretion. This inhibition subsequently impedes phagocyte metabolic transition from oxidative phosphorylation to glycolysis, ultimately suppressing cytokine secretion^94^ (Fig. 3B, b). However, the mechanism of action of the capsules is still unclear, and the capsules’ direct mediation of immune cell responses remains to be explored.

## Niche adaptation

Capsules frequently constitute the outermost layer of cells and facilitate direct interaction between bacteria and their environment, thus influencing bacterial adaptation to new niches. Species encoding capsule, particularly environmental bacteria and facultative pathogens with multiple capsule genes, exhibit higher levels of genetic diversification than their counterparts, contributing to broader environmental adaptability^95^.

During the invasion of a host by pathogenic bacteria, these organisms encounter significant environmental changes, including low pH, elevated temperature, reduced oxygen pressure, and altered osmotic pressure. When pathogenic bacteria invade the epithelial barrier and enter the bloodstream, they are exposed to a 0.15 M sodium chloride osmotic pressure, triggering prioritized CPS synthesis^96^. Upon entering the bloodstream and invading deeper tissues and organs, bacteria encounter the challenge of low oxygen pressure in these environments. In *S. pneumoniae*, CPS synthesis is reduced under hyperoxic conditions compared to hypoxic growth, a phenomenon linked to CpsB phosphatase activity, not CpsD phosphorylation levels^97^. Considering *S. pneumoniae*’s growth environment, its benign colonization in high-oxygen nasopharyngeal areas leads to decreased capsule synthesis. Upon transfer to host defense sites with lower oxygen levels, an increase in capsule synthesis is observed^18^. A similar phenomenon is observed in *S. aureus*, where capsule synthesis in three serotypes is inhibited in environments supplemented with 1%-5% CO_2_^98^. Further research indicated that CO_2_ impedes the transcription of the *cap* gene^99^. However, it is important to note that higher oxygen concentrations are not more beneficial. In *S. pneumoniae*, high oxygen concentrations regulate CpsB phosphatase activity, inhibiting CpsD phosphorylation. Impaired CPS regulation due to tyrosine phosphorylation in CpsD affects *S. pneumoniae*’s capacity to transition from the lungs to the bloodstream^100^. Furthermore, factors such as iron-limited culture^101^, acidic conditions^102,103^, and the nutrient richness^104^ of the environment also play crucial roles in capsule synthesis.

The influence of capsules presence on bacterial adaptation and its role in strain evolution were not empirically confirmed until Nucci et al. conducted the first relevant evolutionary study^105^. In an evolutionary experiment spanning 675 generations (102 days) with three phylogenetically distant strains of *K. pneumoniae*, Nucci et al. discovered that both capsulated and non-capsulated populations possessed a competitive edge over their progenitor strains, with average fitness increases of 58% and 36%, respectively. This finding suggests that capsules play a significant role in enhancing the average fitness of populations^105^. The presence of capsules in adapting populations influences phenotypic changes significantly. Evolved capsulated bacteria exhibit an increase in or the emergence of a hypermucoidy phenotype (HMP), while non-capsulated populations adapt through higher population yields, enhanced surface polysaccharides, and biofilm formation^105^. The competitive advantage of the HMP has been demonstrated in bacteria and fungi, with HMP preventing predation by amoeba or bacteria on *Klebsiella*^106,107^. *Cryptococcus neoformans* develops resistance to amoeba by increasing its capsules size^108^. Furthermore, *E. coli* exhibits increased mucoidy when interacting with macrophages or predatory bacteria, suggesting that this phenotype is advantageous outside the host^109,110^. The research by Nucci et al. suggests that capsulated and non-capsulated populations adapt to niches via distinct pathways. Capsulated strains frequently demonstrate genetic mutations that directly impact capsule synthesis^105^. So, how do non-capsulated cells adapt to the new environment? Firstly, non-capsulated strains adapt through increased production of alternative extracellular polysaccharides on the cell surface, facilitated by Wzi (a functional lectin-binding protein), thereby mimicking capsule functionality^111,112^. Secondly, most non-capsulated clones accumulate mutations in the capsule’s regulatory elements, reducing in the expression cost of other genes within the operon^113^. This reduction in capsule expression may confer an advantage when capsules are regained through horizontal gene transfer. Furthermore, this can result in capsule swapping, expressing a novel serotype with a different biochemical composition among strains with similar chemical compositions^113,114^. Ultimately, the co-adaptation of bacterial populations, both encapsulated and non-encapsulated, leads to a more complex population structure and an increase in cellular interactions^115,116^.

### Other functions

Capsules are known for their contribution to bacterial adhesion and anti-phagocytosis, so these two properties have been most widely studied. With the increasingly in-depth study of capsules, other virulence potentials have gained attention.

Presently, the mortality and morbidity rates linked to bacterial meningitis remain alarmingly high. Earlier studies have reported capsules’ role in enabling bacteria to breach the blood-brain barrier (BBB). *E. coli* with the K1 CPS is particularly prominent among isolates that cause neonatal meningitis. Investigations have revealed that microbial elements, including the K1 capsule, are crucial for the invasiveness of *E. coli*. The K1-cps locus is present in a quarter of bloodstream infection isolates and has independently emerged in at least four ExPEC phylogroups over the last 500 years^34^. Furthermore, the K1 capsule aids in the invasion of brain microvascular endothelial cells (BMEC) by *E. coli*^117^. Unlike other meningitis-causing bacteria like Group B *Streptococci* (GBS)^118^, *E. coli* K1’s invasion does not compromise the integrity of the cell monolayer structure. *E. coli* K1 invades BMEC via a zipper-like mechanism and travels through enclosed vacuoles^119^. Employing a reverse-oriented Transwell filter system with porcine choroid plexus epithelial cells (PCPEC), Tenenbaum et al. explored the process of bacterial invasion and movement from the basolateral (blood) aspect to the apical (cerebrospinal fluid) aspect, a novel approach in this field. Their findings suggest that *S. suis* translocation through PCPEC could be regulated by capsule-derived signals dependent on the lipid kinase phosphatidylinositol 3-kinase pathway (Fig. 3C)^120^. However, there exist conflicting research findings indicating that capsular presence may in fact attenuate bacterial virulence. According to Gendrin et al., GBS strains enhance their intracellular survival and propagation by abandoning their capsule. This mechanism does not involve concealment within macrophages (also called the “Trojan horse” mechanism^121,122^). Concurrently, there is an observed increase in the permeability of the BBB associated with GBS^123^.

The contribution of capsules to bacterial drug resistance is well-documented in scientific literature. In *A. baumannii*, antibiotics induce stress, leading to increased transcription of the K locus genes (responsible for capsule biosynthesis) via the BfmRS two-component regulatory system^124^. This sequence of events triggers capsule synthesis, which confers resistance to chloramphenicol and erythromycin (Fig. 3D, a). In High-alcohol-producing *K. pneumoniae* (HiAlc Kpn), glucose inhibits the expression of *crp* and enhances CPS production, thereby increasing the strain’s drug resistance^125^. Furthermore, several studies have demonstrated that externally added capsules can bind antimicrobial peptides (AMPs), potentially blocking their entry into the cell and diminishing the efficacy of polymyxin (Fig. 3D, b)^126,127^. When the capsule functions naturally, attached to the outer membrane, it increases antimicrobials’ concentration near the cell (Fig. 3D, c)^128^. This elevated concentration of antimicrobial peptides benefits agents like colistin, which disrupt the outer cell membrane and induce cell lysis by binding to lipopolysaccharides and phospholipids^129^. Gendrin et al. noted that reducing capsule density could enhance antibiotic evasion in GBS^123^. Given the limited research, the contribution of the capsule to bacterial antibiotic resistance persists as a debated topic, and the investigation into whether this role is specific to particular antibiotic species remains imperative.

## Regulation mechanisms

### Two-component systems regulate capsules

Two-component systems (TCS) represent a predominant mechanism in bacterial signal transduction^130^. In bacteria, TCS plays a pivotal role in gene regulation, responding to environmental changes. These protein families are implicated in adapting to diverse stress conditions and in essential cellular pathways^131^.

**Fig. 4:**
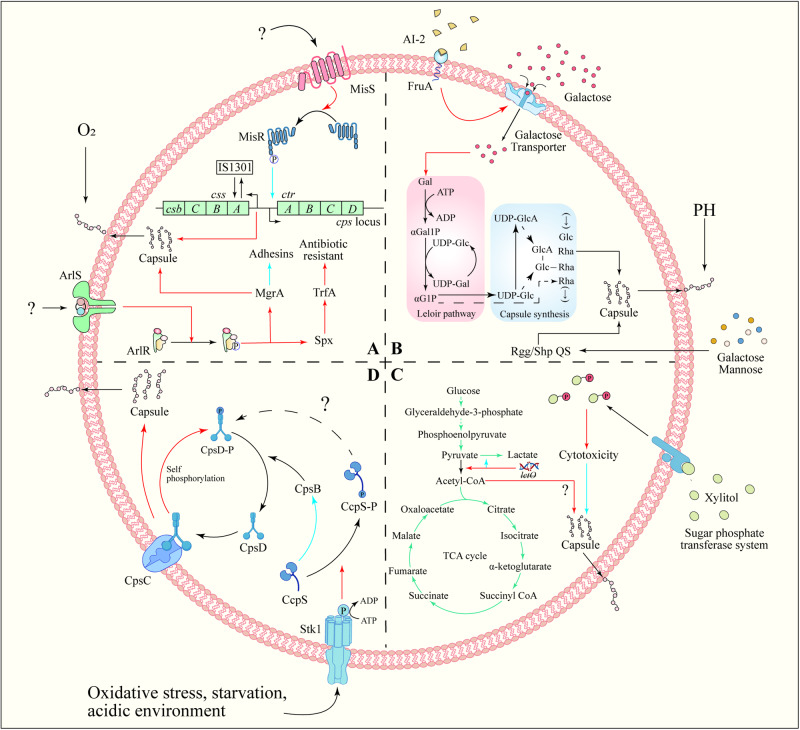
Capsule regulation mechanisms. **A** TCS regulation of capsule synthesis. TCS often regulates capsule biosynthesis by directly binding to the promoters of capsule synthesis genes, thereby responding to environmental changes. The ArlRS TCS of *S. aureus* can directly activate the global transcription factors MgrA and Spx expression, thereby regulating capsule synthesis and antibiotic resistance. *N. meningitidis* MisR/MisS TCS can negatively regulate the production of CPS by directly binding to the promoter region. **B** QS regulation of capsule synthesis. AI-2 regulates the phosphotransferase system on the membrane surface to limit the intake of galactose, ultimately affecting the synthesis of CPS. **C** Metabolic regulation of capsule synthesis. The synthesis of bacterial capsule is related to the fate of pyruvate. Pyruvate is converted into acetyl-CoA and enters the TCA cycle, which is beneficial to capsule synthesis. Therefore, inhibiting the conversion of pyruvate to lactic acid can promote the synthesis of bacterial capsule. In addition, excessive xylitol intake will stimulate the phosphotransferase system on the cell membrane, and phosphorylated xylitol impairs capsule synthesis and ultimately affects bacterial adhesion. **D** External environmental conditions promote Stk1/Stp1 system phosphorylate CcpS, thus relieving the inhibition of CcpS on CpsB. CpsB assists the dephosphorylation of phosphorylated CpsD (CpsD-P). Finally, CpsD binds to CpsC again and promotes CPS synthesis. Positive regulation is indicated by red arrow, and negative regulation by blue arrow.

The *cps* locus promoter in *Neisseria meningitidis* is situated in the intergenic region between biosynthesis and the conserved envelope transport operon. The MisR/MisS TCS negatively regulates CPS production by directly binding to the promoter region (Fig. 4A)^132^. In GAS, the CsrRS TCS as a regulator of capsule production. This system comprises the loci *csrR* and *csrS* (also known as *covR* and *covS*)^133^. While the inactivation of *csrR* did not alter M protein expression or hemolytic activity, it resulted in a sixfold increase in capsule production, whereas subsequent studies showed that the system influences the expression of additional virulence factors, including streptokinase (*ska*), mitogenic factor (*speMF*) and cysteine protease (*speB*)^28^. The RstAB TCS positively regulates CPS synthesis, aiding *Photobacterium damselae* in evading fish host cells’ defense^134^. ArlR directly activates the expression of global transcription factors MgrA and Spx in *S. aureus*^135^ (Fig. 4A), influencing capsule synthesis genes, wall-anchored adhesins (*ebh*, *sdrD*), cell wall remodeling genes (*lytN*, *ddh*) and anaerobic metabolism genes (*adhE*, *pflA*, *nrdDG*). This activation promotes capsule synthesis and impacts the TrfA protein, a component of the Clp proteasome complex, which plays a role in resisting cell wall-targeting antibiotics, thereby enhancing antibiotic resistance^136,137^.

In summary, most TCS are recognized as key molecular mechanisms regulating bacterial capsule synthesis, and the understanding of their interrelationships is becoming increasingly comprehensive.

### Quorum sensing regulates capsules

Quorum sensing (QS) represents a form of inter-bacterial ‘communication’^73^. In this process, bacteria synthesize and detect extracellular signaling molecules, termed auto-inducers (AI), leading to either the activation of regulatory proteins or the suppression of specific gene expression. This intricate system plays a crucial role in bacterial coordination and collective behavior. This process allows for the control of the physiological characteristics of microbial populations, including traits like motility, biofilm formation, immunosuppression, and nutrient utilization^138,139^. Studies have indicated that QS-regulated genes constitute 10% and 20% of the entire genome^140,141^. As research on QS deepens, more attention is being directed toward understanding the relationship between QS and capsules.

FruA, a fructose-specific phosphotransferase system component in *S. pneumoniae*, can sense AI-2. This sensing mechanism leads to the up-regulation of the galactose ABC transporter and the Leloir pathway (Fig. 4B). Subsequently, it increases CPS synthesis, resulting in a high virulence phenotype^142^. Rgg/Shp144 and Rgg/Shp939 were proved to be another QS of *S. pneumoniae* by Zhi et al. The Rgg/Shp QS includes Rgg proteins (alternatively termed Gad or Mut), which are part of a conserved group of independent transcriptional regulators, and short hydrophobic peptides (Shp)^143,144^. Rgg/Shp1517 QS is necessary for *S. pneumoniae* to use galactose and mannose. However it is a negative regulator of capsular expression, which may involve binding Rgg to the capsular locus promoter (Fig. 4B)^145^. Additionally, the regulation of CPS by Rgg/Shp QS varies depending on the response to specific sugars. Rgg144 and Rgg939 were most significantly induced by mannose, followed by galactose, whereas Rgg1518 was primarily stimulated by galactose^146^. Furthermore, other quorum sensing systems have also been reported to be associated with capsule synthesis. For example, GtaR/I QS is involved in regulating *Rhodobacter capsulatus* (RcGTA) growth and capsules’ formation, and the capsules also play a role in RcGTA adhesion as a receptor^147^. Agr (accessory gene regulator) QS positively regulates the production of type-5 capsular polysaccharide (CP5) in *S. aureus*, enhancing adhesion to inner epidermal cells (EC)^82^.

### Metabolic activity regulates capsules

Capsules are crucial in bacterial invasive infections, acting as an energy-consuming virulence factor. Bacteria frequently utilize carbohydrates via glycolysis and other central carbon metabolism (CCM) to support the energy demands of capsule synthesis^104^. CCM encompasses enzymatic reactions converting carbon into energy, including glycolysis, the tricarboxylic acid (TCA) cycle, the gluconeogenesis, the pentose phosphate pathway (PPP), the glyoxylate shunt, the γ-aminobutyric acid (GABA) shunt, and the methylcitrate cycle^148^. As a central component of material metabolism, the TCA cycle plays an irreplaceable role in the life process of organisms. Pyruvate from glycolysis is converted to acetyl-CoA by pyruvate dehydrogenase, initiating the TCA cycle and providing energy for cells. Following the simultaneous deletion of *spxB* (pyruvate oxidase) and *lctO* (lactate dehydrogenase), acetyl-CoA and capsule production were restored in *S. pneumoniae* type 4, though the underlying mechanism remains elusive^149^. The prevailing hypothesis posits that the absence of lactate dehydrogenase leads to increased lactic acid levels, inhibiting lactate dehydrogenase activity. This inhibition facilitates bacterial capture of pyruvate, thus enhancing the conversion of pyruvate to acetyl-CoA (Fig. 4C). Although *S. aureus* possesses pyruvate dehydrogenase, it directly converts pyruvate to acetic acid^150^. However, the impact of this conversion on acetyl-CoA and capsule synthesis remains unknown. Excessive sugar intake is deleterious, given that the glucose phosphotransferase system swiftly transports xylitol into cells and phosphorylates it, while the accumulation of excessive xylitol phosphate can exert toxic efforts on *S. pneumoniae*, impairing CPS production and diminishing adhesion to nasopharyngeal cells (Fig. 4C)^151^. CPS in *S. aureus* serotype 5 was eliminated in the presence of glucose, but this did not affect CPS synthesis in the *ccpA* (coding catabolite control protein A) deletion strain MST14 serotype 5. Seidl et al. found that *cap* operon expression in *S. aureus* serotype 5 was significantly lower compared to MST14, yet no prominent catabolite-responsive elements (CREs) were identified in the *cap* operon’s genomic region, suggesting an indirect effect of CcpA on *cap* transcription^152^.

Additionally, a complex relationship exists between capsule types and metabolic cost. For instance, capsule exchange in *S. pneumoniae* may result in diminished fitness or viability, a consequence modulated by the carbon content and CPS charge of each polysaccharide^153^. Hathaway et al. compared growth phenotypes of *S. pneumoniae* across different capsular serotypes, finding that strains with a lower metabolic burden exhibited growth advantages^104^. Schipper et al. examined the impact of meningococcal CPS structure on the lethality of zebrafish embryos and neutrophil consumption post-infection. They observed a close relationship between the CPS structure and the carbon number in each polysaccharide repeat unit. Consequently, the variation in virulence among different capsule types may stem from metabolic cost differences rather than molecular interactions with host immune components^154^.

### Other regulation mechanisms

Tyrosine phosphorylation, initially viewed as crucial in eukaryotic regulation^155^, is now recognized as a critical factor in bacterial physiology^156^. Although phosphorylation is a longstanding recognized posttranslational regulatory mechanism in bacteria, the significance of tyrosine phosphorylation was highlighted with the discovery of a tyrosine-phosphorylated protein in *Acinetobacter johnsonii*^157^. The cocci bacteria encode the first four genes, *cpsABCD*, in the *cps* locus, a sequence broadly conserved across species^18^. The *cpsA* gene encodes LytR-Cps2A-Psr (LCP) protein, which is believed to conjugate CPS to peptidoglycan (PG)^158^. The *cpsB*, *cpsC*, and *cpsD* genes form a tyrosine phosphoregulatory system controlling CPS assembly machinery^159,160^. CpsC is essential for CpsD’s tyrosine phosphorylation. When CpsD self-phosphorylates (utilizing bound ATP), the resulting tyrosine phosphorylated CpsD (CpsD-p) dissociates from CpsC, reducing CPS production. CpsB assists in CpsD-p dephosphorylation, facilitating its interaction with CpsC, leading to an accelerated rate of CPS biosynthesis/polymerization^161,162^. The CpsBCD bacterial tyrosine kinase system responds to environmental changes (e.g., oxygen content^18^), with the mechanism clarified by Tang et al.’s study in *S. suis*, enhancing understanding of the relationship between CpsBCD and signal transduction. CcpS, a protein regulating phosphatase CpsB’s activity, links the Stk1/Stp1 system (a serine/threonine kinase system controlling bacterial phosphosignaling) with the Wzx-Wzy pathway in bacteria. Stk1/Stp1 specially mediates Thr-phosphorylation of the CcpS protein. Non-phosphorylated CcpS can inhibit CpsB-catalyzed dephosphorylation of CpsD-P in vivo, leading to abnormal CPS synthesis in *S. suis*^163^ (Fig. 4D).

The second messenger cyclic AMP (cAMP), small RNA, and iron-acquisition systems are also found to be associated with capsule synthesis. Cyclic-3’,5’-adenosine monophosphate (cAMP) is a ubiquitous second messenger, orchestrating essential processes in bacteria and eukaryotes^164^. *K. pneumoniae* regulates CPS production through cAMP-dependent carbon catabolite repression (CCR), enhancing protection from serum killing and phagocytosis and modifying oxidative stress resistance, improving phagosome survival^165^. In *Vibrio parahaemolyticus*, AI-2 QS controls the capsular synthesis and bacterial aggregation through self-inducing signals (S signals) affecting c-di-GMP levels^166^. Small RNAs (sRNAs), such as rss04 and rss03, are critical regulators of bacterial virulence, inhibiting CPS production after *S. suis* enters the brain, exacerbating the inflammatory response, and promoting meningitis^167^. The iron uptake system, a crucial regulatory mechanism in bacteria, has become a focal point of research. Bacteria stringently regulate iron transport and storage via Fur (ferric uptake regulator) to maintain iron homeostasis. Under iron-replete conditions, dimeric Fur complexed with Fe(II) binds to a 19 bp consensus DNA sequence in the promoters of iron uptake genes, inhibiting their transcription^168^. In *K. pneumoniae*, Fur suppresses CPS biosynthesis by inhibiting RmpA and RcsA. Interestingly, sRNA also plays a role in these regulatory activities^169^. sRNA RyhB activates the transcription of *orf1* and *orf16*, components of the *cps* gene cluster open reading frames (ORFs)), independently of RmpA and RcsA^170^. However, since *K. pneumoniae* requires CPS for survival in the host, other positive regulatory systems responding to external iron influence CPS biosynthesis. IscR, a protein harboring a [2Fe-2S] cluster and encoded by the first gene of the *iscRSUA* operon^171^, orchestrates the regulation of genes engaged in diverse cellular processes that respond to environmental stimuli such as oxidative stress and iron^172^. With the [2Fe-2S] clusters, IscR’s DNA binding specificity is broadened, enabling holo-IscR to interact with both type 1 and type 2 IscR box^173^, positively influencing CPS biosynthesis^174^.

## Outlook

Given the substantial immunomodulatory characteristics of CPS, they have garnered considerable attention in vaccine development. Extensive research over the years has established the efficacy of vaccines based on polyvalent pneumococcal polysaccharides. This focus aligns with CPS’s ability to modulate immune responses, underscoring its potential in preventive healthcare strategies. Mutagenesis-induced removal of CPS reveals antigenic cell wall proteins usually obscured by the dense capsular shell. The nonencapsulated mutant is anticipated to elicit a more potent immune response. Although the capsule’s structural diversity, biosynthesis, and immunogenicity have been extensively studied, further research is required on their role in pathogen adhesion and regulatory mechanisms. Secondly, research on immune evasion predominantly centers on how capsules mask surface antigens of pathogenic bacteria. Studies on the capsule’s active mediation of host immunity (e.g., *F. tularensis*^94^) remain limited. Conducting further research will undoubtedly deepen our understanding of the capsule’s biological functions and aid in developing treatment strategies. Additionally, bacteria can express multiple capsule types concurrently^65,175^. This capability of co-expression broadens the bacteria’s range of environmental adaptabilities, including enhanced adhesion and virulence, thereby facilitating their ecological transition towards host colonization and pathogenesis^176^. Therefore, in the future, it is necessary to study in detail the mechanisms that lead to the acquisition of multiple capsules.
